# Interaction of Alpha-Synuclein With Lipids: Mitochondrial Cardiolipin as a Critical Player in the Pathogenesis of Parkinson’s Disease

**DOI:** 10.3389/fnins.2020.578993

**Published:** 2020-10-06

**Authors:** Valentina Gilmozzi, Giovanna Gentile, Maria Paulina Castelo Rueda, Andrew A. Hicks, Peter P. Pramstaller, Alessandra Zanon, Martin Lévesque, Irene Pichler

**Affiliations:** ^1^Institute for Biomedicine, Eurac Research, Affiliated Institute of the University of Lübeck, Bolzano, Italy; ^2^Department of Neurology, University of Lübeck, Lübeck, Germany; ^3^Department of Psychiatry and Neurosciences, Cervo Brain Research Centre, Université Laval, Quebec, QC, Canada

**Keywords:** alpha-synuclein, Parkinson’s disease, lipids, cardiolipin, aggregation

## Abstract

Alpha-Synuclein (α-Syn) is a central protein in the pathogenesis of synucleinopathies, a group of neurodegenerative disorders including Parkinson’s disease (PD). Although its role in neurotransmission is well established, the precise role of this protein in disease pathogenesis is still not fully understood. It is, however, widely regarded to be associated with the misfolding and accumulation of toxic intracellular aggregates. In fact, α-Syn is the most abundant protein component of Lewy bodies and Lewy neurites, which are also characterized by a high lipid content. Lipids, the main constituents of cellular membranes, have been implicated in many aspects of PD-related processes. α-Syn interacts with membrane phospholipids and free fatty acids via its N-terminal domain, and altered lipid-protein complexes might enhance both its binding to synaptic and mitochondrial membranes and its oligomerization. Several studies have highlighted a specific interaction of α-Syn with the phospholipid cardiolipin (CL), a major constituent of mitochondrial membranes. By interacting with CL, α-Syn is able to disrupt mitochondrial membrane integrity, leading to mitochondrial dysfunction. Additionally, externalized CL is able to facilitate the refolding of toxic α-Syn species at the outer mitochondrial membrane. In this review, we discuss how α-Syn/lipid interactions, in particular the α-Syn/CL interaction at the mitochondrial membrane, may affect α-Syn aggregation and mitochondrial dysfunction and may thus represent an important mechanism in the pathogenesis of PD.

## Introduction

Parkinson’s disease (PD) is a neurodegenerative disorder caused by the loss of dopaminergic neurons in the *substantia nigra pars compacta*. It is part of the synucleinopathies, diseases whose hallmark is represented by Lewy bodies (LB), which are characterized by alpha-synuclein (α-Syn) inclusions (Spillantini et al., 1997). α-Syn is an intrinsically disordered protein (Weinreb et al., 1996) and is highly expressed in neurons (Nakajo et al., 1990; Iwai et al., 1995). Although its physiological function is still not completely understood, α-Syn is believed to be involved in multiple cellular processes including vesicular trafficking, clustering of synaptic vesicles, maintaining synaptic vesicle pools, and neuronal transmission (reviewed by Bendor et al., 2013). α-Syn is also implicated in endoplasmic reticulum stress and mitochondrial dysfunction, impacting the electron transport chain, and mitochondrial dynamics (reviewed by Wong and Krainc, 2017).

α-Syn is encoded by the *SNCA* gene (Shibasaki et al., 1995) and consists of 140 amino acid residues resulting in a molecular weight of about 14 kDa. Its primary sequence is usually divided into three distinct regions: (1) the positively charged N-terminal region (residues 1–60), which contains seven imperfect 11-amino acid repeats with a conserved sequence (KTKEGV) and adopts a helical structure (residues 3–94; Ulmer et al., 2005) upon binding to lipids (Georgieva et al., 2008), (2) the hydrophobic core (residues 61–95), also termed as non-amyloid-beta component (NAC) region, showing a high propensity for α-Syn aggregation (Rodriguez et al., 2015), and (3) the highly acidic C-terminal region (residues 96–140), which remains unstructured even in the presence of lipids (Ulmer et al., 2005).

Under physiological conditions, α-Syn exists both as an intrinsically disordered form in the cytosol (Weinreb et al., 1996) and a lipid membrane-bound helical conformation (Davidson et al., 1998). Processes underlying misfolding and aggregation of α-Syn are generally thought to have a central role in the pathogenesis of PD (reviewed by Eschbach and Danzer, 2014). However, how α-Syn converts to form insoluble beta-sheets by recruiting monomers to form protofibrils and amyloid fibrils is still not fully defined (reviewed by Meade et al., 2019). The process of fibril formation might start by forming an aggregation nucleus from monomers (nucleation) followed by a fibril growth phase (elongation of the nuclei) by monomer addition, and a third phase, in which monomers and aggregates are in equilibrium (Wood et al., 1999; Li et al., 2009). Among the different α-Syn species, β-sheet rich amyloid fibrils are thought to be the basis of LBs (Shults, 2006; Araki et al., 2015), although there is mounting evidence that oligomers are also a potent toxic species of α-Syn, able to induce neurodegeneration in dopaminergic neurons (Conway et al., 2000; Karpinar et al., 2009; Winner et al., 2011). A recent study by Shahmoradian et al. report “a non-fibrillar form of α-Syn” in the analyzed LBs of patients (Shahmoradian et al., 2019) but more studies including a larger set of samples will be needed to confirm these findings (reviewed by Lashuel, 2020).

The modulation of α-Syn folding depends on both intrinsic and extrinsic factors. On one hand, there is the influence of point mutations and multiplications in the *SNCA* gene as well as post-translational modifications, and on the other hand, there is the environment, in which the protein coexists with other molecules and ions. Modifications to the primary structure by disease-causing point mutations (A53T, A30P, E46K, H50Q, A18T, A29S, G51D, A53E, A53V; Polymeropoulos et al., 1997; Kruger et al., 1998; Zarranz et al., 2004; Appel-Cresswell et al., 2013; Hoffman-Zacharska et al., 2013; Lesage et al., 2013; Pasanen et al., 2014; Yoshino et al., 2017) or genomic multiplications (*SNCA* duplication or triplication; Singleton et al., 2003; Chartier-Harlin et al., 2004) cause α-Syn protein dyshomeostasis and eventually lead to α-Syn aggregation. Indeed, changes in the concentration of α-Syn, and its folding state, combined with the formation of multimeric species, define the transition toward pathological conditions (reviewed by Bobela et al., 2015). The three distinct regions of α-Syn contribute differently to the protein conformation. Long-range contacts between C-terminal and N-terminal as well as C-terminal and NAC elements may act to isolate the hydrophobic region of the protein, inhibiting spontaneous α-Syn oligomerization (Bertoncini et al., 2005; Cho et al., 2009). In addition, the N-terminal region contains two sequence motifs named P1 (residues 36–42) and P2 (preNAC, residues 45–57), which show significant aggregation propensity and are important for α-Syn-mediated membrane fusion (Doherty et al., 2020). Notably, the preNAC region contains six of the nine known missense mutations (G51D, A53V, A53E, H50Q, E46K, A53T) causing loss-of-function and/or gain-of-toxic function leading to PD onset (Fares et al., 2014; Mohite et al., 2018; Picillo et al., 2018; Boyer et al., 2019, 2020; Sun et al., 2020). The aggregation status of α-Syn is also influenced by diverse post-translational modifications, including phosphorylation mainly at serine 129 (Ser129-P; reviewed by Zhang et al., 2019), which has emerged as a defining hallmark of PD and other synucleinopathies. α-Syn structure, aggregation, and membrane-binding properties are also impacted by environmental stimuli, such as redox-active metals like copper and iron (reviewed by Carboni and Lingor, 2015).

In this review, we discuss how α-Syn-membrane interactions, stimulated by phospholipids and fatty acids (FAs), may affect α-Syn aggregation. In particular, we outline the α-Syn/cardiolipin (CL) interaction at the mitochondrial membrane and its consequences on mitochondrial and cellular function, which might represent an important pathological mechanism in PD.

## Alpha-Synuclein Interaction With Membrane Phospholipids

Neurodegenerative diseases including PD are associated with lipid dyshomeostasis, and defective lipid signaling affects dopaminergic neuron-specific signaling cascades like neurotransmission and receptor activation (Klemann et al., 2017). Moreover, it has been recently shown that excess α-Syn correlates with alterations in lipid pathways (Fanning et al., 2019) and that LBs are rich in membranous lipids that originate from vesicles and fragmented organelles (Shahmoradian et al., 2019).

For the study of α-Syn interaction with membrane lipids, membranous fractions, synaptic vesicles, intact cells, and various synthetic model membranes are used (reviewed by Beyer, 2007). There is a general consensus that the repeat motifs characterizing the N-terminal region enable α-Syn to transiently and dynamically interact with target membranes (reviewed by Bendor et al., 2013). In physiological conditions, monomeric α-Syn switches rapidly between highly curved membranes and cytosol (Fortin et al., 2005) and following membrane binding, α-Syn undergoes a transient conversion from random coils to a helical structure in its N-terminal region. These amphipathic helices are promoted by positively charged lysine residues in the hydrophilic part of the repeat motif that interact with negatively charged phospholipid head groups, and by hydrophobic amino acids of the α-Syn repeat motif, which interact with the fatty acyl chains of membrane lipids (Stockl et al., 2008). α-Syn preferentially binds to membranes with a specific lipid composition, characterized by negative charge and high curvature (Middleton and Rhoades, 2010), emphasizing the importance of electrostatic interactions with the positively charged N-terminus and the contribution of hydrophobic (van der Waals) interactions with the fatty acyl chains, respectively. In addition, small vesicles with loosely packed membranes facilitate the binding of α-Syn (Nuscher et al., 2004). Notably, a recent study suggests a soluble helical multimer formation of α-Syn when released from the membrane (Rovere et al., 2018).

The transient interaction between α-Syn and membranes can be stabilized by increasing the hydrophobicity in the membrane-inserted part of the α-Syn amphipathic helix. One way to augment α-Syn hydrophobicity is to induce point mutations (KLKEGV and KTKEIV mutants, changed in six or seven repeats of the core motif, respectively) by introducing non-polar amino acids in the repeat motif (Dettmer et al., 2017). These membrane-enriched mutations were shown to lead to insoluble α-Syn fractions and round cytoplasmic inclusions, characterized by clusters of vesicles, inducing acute neurotoxicity (Dettmer et al., 2015). The familial PD mutation E46K (KTKKGV in repeat 4 of the KTKEGV motif) also leads to an increased α-Syn/membrane interaction, possibly due to additional positive charges in the hydrophilic half of the amphipathic helix (Perlmutter et al., 2009). However, reduced α-Syn/membrane interaction has also been reported to promote α-Syn aggregation (Burre et al., 2015).

Overall, altered binding of α-Syn to vesicles and other phospholipid membranes could lead to an imbalance between the cytosolic and membrane-bound α-Syn forms as well as between monomeric and multimeric forms, resulting in an early stage of α-Syn accumulation.

## Alpha-Synuclein Interaction With Fatty Acids

Under physiological conditions, α-Syn controls the oxidative homeostasis of the intracellular environment, protecting free FAs from oxidation and maintaining their appropriate levels (Sharon et al., 2003b; De Franceschi et al., 2017). Conversely, it has been suggested that unsaturated fatty acids (UFAs) act as scavengers by reacting with peroxynitrite and diminishing the amount of oxidant interacting with α-Syn (Trostchansky et al., 2006). Several studies suggest that α-Syn interacts with FAs in the neuronal cytoplasm, which might trigger the formation of lipid-associated oligomers (Sharon et al., 2001, 2003a; Lucke et al., 2006). Furthermore, overexpression of the brain-specific fatty acid-binding protein 3 (FABP3), which increases the cellular uptake of arachidonic acid (AA), was shown to trigger α-Syn oligomerization (Shioda et al., 2014). As for the interaction with membranes, the N-terminal region of α-Syn is also essential for the binding to FAs and the FA-induced oligomerization of the protein (Karube et al., 2008). A53T mutant α-Syn binds more tightly to lipid droplets (and membranes) than wild-type α-Syn, whereas A30P mutant α-Syn remains primarily cytosolic (Cole et al., 2002), showing that pathological mutations in the *SNCA* gene influence the binding of the protein to FAs. Notably, the cytoplasmic aggregates resultant of artificially generated α-Syn mutants in the KTKEGV motif were often observed in close proximity to lipid droplets (Dettmer et al., 2017).

Based on their degree of saturation, FAs are classified as saturated (SFAs), monounsaturated (MUFAs), and polyunsaturated (PUFAs). The degree of saturation influences the interaction with α-Syn and the level of oligomerization. Indeed, the exposure of neuronal cells stably expressing wildtype human α-Syn to MUFAs and SFAs did either not affect or decrease the levels of α-Syn oligomers, whereas PUFAs rapidly and dramatically increased α-Syn oligomer levels (Sharon et al., 2003a). Therefore, the more unsaturated the FAs are, the more effectively they seem to promote the appearance of α-Syn oligomers. PUFAs exert their effects on α-Syn when organized as either a micellar (free FAs) or vesicular (phospholipid) surface (Perrin et al., 2001). Lipid peroxidation byproducts derived from PUFAs might modify α-Syn, which could further contribute to increased oligomer formation and toxicity (reviewed by Shamoto-Nagai et al., 2018). However, the PUFA AA might also promote the formation of helical α-Syn multimers, which are resistant to fibril formation (Iljina et al., 2016). PUFA levels, specifically docosahexaenoic acid (DHA), were shown to be increased in the cytosolic fraction of frontal cortex of PD patients (Sharon et al., 2003b). Additional studies confirmed increased DHA levels in frontal cortex (Dalfo et al., 2005; Fabelo et al., 2011), while DHA and AA content were decreased in frontal cortex lipid rafts, ordered membrane regions, from PD patients (Fabelo et al., 2011). Furthermore, increased or unchanged PUFA levels in the anterior cingulate cortex and the occipital cortex of PD patients were reported, respectively (Abbott et al., 2015). These data point to increased PUFA levels in whole cortical tissues and reduced levels in lipid rafts. The relationship between MUFAs and α-Syn is underlined by recent studies showing that the inhibition of stearoyl-CoA desaturase (SCD), a key enzyme in MUFA biosynthesis, is able to reduce α-Syn cytotoxicity by increasing physiological α-Syn multimerization and solubility and decreasing α-Syn phosphorylation on Ser129-P (Vincent et al., 2018; Fanning et al., 2019; Imberdis et al., 2019). Levels of MUFAs, specifically oleic acid levels, were found to be increased in α-Syn overexpression models, and excess oleic acid led to the formation of α-Syn inclusions (Fanning et al., 2019).

## Alpha-Synuclein Interaction With the Mitochondrial Phospholipid Cardiolipin

Mitochondria are complex, dynamic organelles, which are surrounded by a unique double membrane, consisting of outer (OMM) and inner (IMM) mitochondrial membranes, each comprising a lipid bilayer, and mitochondrial contact sites, regions of close apposition between OMM and IMM. They have a prominent role in energy metabolism and are involved in a plethora of additional key cellular processes, including oxidative stress response, calcium homeostasis, and cell death pathways. A critical role for mitochondrial dysfunction in the pathophysiology of PD has been established based on observations that mitochondrial toxins can cause parkinsonism in humans and animal models and that mitochondrial complex I activity was reduced in the *substantia nigra* of patients with sporadic PD (reviewed by Camilleri and Vassallo, 2014). Furthermore, the majority of genes linked to monogenic forms of PD are associated with mitochondrial dysfunction (reviewed by Park et al., 2018).

Recent data collected in *postmortem* human brain tissue from PD brain donors suggests that LB inclusions do not principally contain fibrillar α-Syn as previously thought, but are characterized by crowding of α-Syn, lipids, vesicular structures and fragmented organelles including mitochondria, supporting a key role of damaged organelles in the formation of LBs (Shahmoradian et al., 2019). In fact, Ser129-P α-Syn has been recently found to be primarily located at the mitochondria in neuronal and mouse models of PD as well as *postmortem* brain samples of patients with synucleinopathies (Ludtmann et al., 2018; Ryan et al., 2018; Wang et al., 2019).

**FIGURE 1:**
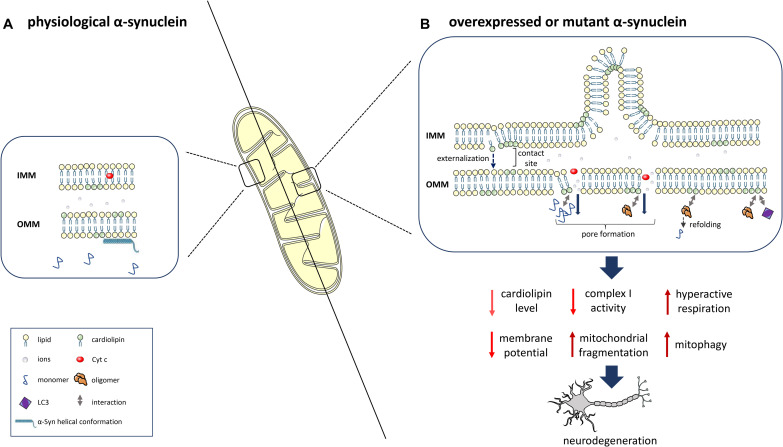
α-Syn interaction with the mitochondrial phospholipid cardiolipin (CL) and downstream consequences. **(A)** At physiological levels, α-Syn binds to mitochondrial membranes in a helical conformation and does not impact CL levels. **(B)** α-Syn overexpression, mutation or knockout determines reduced levels, an altered composition and oxidation state of CL and the translocation of CL from the IMM to the OMM. Prolonged CL exposure leads to excessive mitophagy due to increased binding of CL to LC3. α-Syn also leads to OMM permeabilization due to pore formation, ion leakage, cytochrome c release, destabilization of the electron transport chain complexes and reduced complex I activity/hyperactive respiration. OMM-localized CL facilitates the refolding of α-Syn oligomers and fibrils. This figure was created using elements from Servier Medical Art, which is licensed under a Creative Commons Attribution 3.0 Unported Generic License (https://creativecommons.org/licenses/by/3.0/).

Alpha-Synuclein aggregates might interact with mitochondrial proteins and interfere with their function, as shown for the interaction of post-translationally modified forms of α-Syn (soluble oligomers, dopamine modification, and the phosphomimetic mutant S129E) with TOM20 (translocase of the outer membrane 20), which leads to an impairment of mitochondrial protein import and function (Di Maio et al., 2016). An important role of lipids in the α-Syn/mitochondria interaction is supported by the binding of α-Syn to mitochondrial phospholipids, particularly CL (Cole et al., 2008; Nakamura et al., 2008; Zigoneanu et al., 2012). The interaction is mediated by the N-terminal part of α-Syn, which adopts a helical conformation (Robotta et al., 2012, 2014). Specifically, α-Syn preferentially binds to CL-enriched mitochondrial membrane microdomains (Ramakrishnan et al., 2003; Grey et al., 2011), potentially inducing pore formation and mitochondrial dysfunction (Ghio et al., 2019). Under physiological conditions, α-Syn is localized to the synapse together with a high number of mitochondria (Hollenbeck, 2005) to satisfy the high bioenergetic requirements of dopaminergic neurons (Pacelli et al., 2015). By interacting with CL, α-Syn is targeted to and physically interacts with mitochondrial membranes, thereby potentially disrupting their integrity and inducing mitochondrial dysfunction. This interaction with mitochondrial membranes might therefore represent an important mechanism in the pathogenesis of PD (Figure 1).

The phospholipid CL is considered the hallmark lipid of mitochondria (Pangborn, 1945). In normal conditions, it is primarily located in the IMM, where it makes up 15–20% of the total lipid content (Daum, 1985), with up to 5% abundance in the OMM (Colbeau et al., 1971; de Kroon et al., 1997) and inner-outer membrane contact sites (Kim et al., 2004). Structurally, CL is characterized by two negative charges associated with its two phosphatidic acid residues linked by a glycerol bridge, and four associated fatty acyl chains, which differ in length and saturation (reviewed by Schlame, 2008). CL biosynthesis, assembly, and remodeling of its FAs occurs in the IMM (reviewed by Schlame and Greenberg, 2017), while the translocation of CL to the OMM acts as a starting signal for the apoptotic process through the release of cytochrome c (Cyt c; Petrosillo et al., 2001). CL is also involved in the early phases of the selective removal of injured mitochondria by macroautophagy (mitophagy). The externalization of CL to the OMM was identified as a mitophageal signal, marking mitochondria as damaged, and causing their removal through the interaction with the autophagy protein LC3, which mediates both autophagosome formation and cargo recognition (Chu et al., 2014). In physiological conditions, CL contains a high number of PUFAs, which makes it an easy target of peroxidation with relevant consequences for mitochondrial function (reviewed by Paradies et al., 2009). An abnormal CL content, fatty acyl chain composition, and level of oxidation are associated with a significant decrease of membrane potential and pleiotropic defects in mitochondrial function, including mitochondrial enzyme activities, oxidative phosphorylation, protein import, mitochondrial biogenesis, and mitochondrial membrane morphology and dynamics (reviewed by Paradies et al., 2019). Such CL abnormalities and associated mitochondrial dysfunction have been detected in multiple tissues and a variety of pathological conditions (Monteiro et al., 2013), although a small study including only 12 PD patients did not detect altered CL levels in the *substantia nigra* (Seyfried et al., 2018).

Upon binding to CL, α-Syn monomers adopt an amphipathic helical structure (Robotta et al., 2014; Stefanovic et al., 2014; Ryan et al., 2018), and also the binding of oligomers to bilayers composed of lipid mixtures that mimic the composition of the IMM leads to an increase in helical structures, which was, however, much lower as compared to monomers. Only the monomers in the oligomer facing the bilayer and binding the membrane might adopt a helical conformation, and the interactions between monomers in the oligomer might be too strong to permit further conformational changes upon membrane binding (Stefanovic et al., 2014). Furthermore, α-Syn oligomers seem to be able to grow continuously in distinct domains of membrane mimics (Grey et al., 2011). External application of α-Syn aggregate complexes induced robust mitochondrial membrane permeabilization and triggered the release of Cyt c from isolated mitochondria. These effects on mitochondria, which were more pronounced for mutant α-Syn (A30P and A53T), were dependent on CL (Camilleri et al., 2013). The presence of CL (∼15%) in bilayers with a physiological phospholipid composition that simulates the mitochondrial membrane (IMM and contact sites), was shown to enhance the α-Syn/membrane interaction and the formation of membrane pores by α-Syn oligomers, resulting in mitochondrial swelling and Cyt c release. Notably, the pore forming activity was higher in mitochondrial-like membranes with high CL content as compared to bilayers with a phospholipid composition reflecting synaptic vesicle membranes (Ghio et al., 2019). In line with these data, α-Syn monomers showed a favorable interaction with phosphatidylglycerol vesicles over phosphatidylserine vesicles, including deeper insertion into phosphatidylglycerol membranes resulting in extensive membrane rupture (Hannestad et al., 2020). While phosphatidylserine is found in synaptic vesicle membranes (Lim and Wenk, 2009), phosphatidylglycerol is a precursor for CL synthesis (Stanacev et al., 1967), and as such plays an important role in mitochondrial membranes. The different consequences of α-Syn/membrane interactions depending on their lipid composition suggest their relevance for both physiological function, like vesicle binding, and dysfunction, like mitochondrial membrane disruption. In the study by Hannestad et al. (2020), an initial α-Syn monomer binding accompanied by a rapid clustering of additional monomers causing membrane leakage was suggested, which might be consistent with previous reports of membrane-bound α-Syn being able to seed the aggregation of the protein (Lee et al., 2002; Grey et al., 2011). However, further studies are needed to better characterize membrane-bound α-Syn aggregate species not only by using model membranes but also when bound to membranes *in vivo*.

The selective interaction of α-Syn with CL may be partly explained by electrostatic interactions between the divalent anionic charge from the diphosphatidyl glycerol headgroup of CL and the positively charged lysine residues in the N-terminal repeat domain of α-Syn (Ramakrishnan et al., 2003; Zigoneanu et al., 2012; Shen et al., 2014). However, in the study by Ghio et al. (2019), both the mitochondrial membrane and the synaptic vesicle mimics had the same overall anionic charge allowing to control for an effect of negative charge on pore forming activity. Furthermore, the conical shape of CL induces high intrinsic curvature in CL-enriched microdomains as in the highly curved edges of mitochondrial cristae in the IMM (Elias-Wolff et al., 2019). Lastly, CL decreases the mechanical stability of the membrane, disturbing lipid packing (Unsay et al., 2013), which might explain the propensity of α-Syn oligomers, which show a high affinity for loosely packed membranes (Ouberai et al., 2013), to bind CL-enriched membrane domains.

Overexpression of the N-terminal part of α-Syn causes a decrease of CL levels in dopaminergic MN9D cells and primary cortical neurons (Shen et al., 2014) and mouse models (Gao et al., 2017), compromising mitochondrial membranes and altering mitochondrial function. Overexpression of α-Syn also led to an increased expression of ALCAT1, an acyltransferase involved in the pathological remodeling of CL, whereas ALCAT1 deficiency prevented α-Syn oligomerization and phosphorylation at Ser129 (Song et al., 2019). There is, however, no definitive evidence as to whether the reduction in CL levels/species is located upstream or downstream of the α-Syn interaction with mitochondria. On the other hand, the brain of mice lacking α-Syn (*Snca-/-*) also showed an overall reduction of CL content with an altered CL acyl chain composition, which was associated with a reduced activity of respiratory chain complexes I and III (Ellis et al., 2005). An additional study on *Snca-/-* mice found reduced CL mass and its precursor phosphatidylglycerol (Barcelo-Coblijn et al., 2007), suggesting that the synthesis of CL is altered in the absence α-Syn.

The selective binding of misfolded α-Syn to CL was shown to cause hyperactive mitochondrial respiration in SH-SY5Y neuroblastoma cells (Ugalde et al., 2020). Furthermore, overexpressed wild-type α-Syn as well as A53T and E46K mutants, through the interaction with CL, directly affect mitochondrial membrane dynamics by inducing mitochondrial fragmentation, whereas the A30P α-Syn mutation does not affect mitochondrial morphology (Nakamura et al., 2011). In agreement with these data, A53T and E46K mutant neurons derived from human pluripotent stem cells showed highly fragmented mitochondria, reduced mitochondrial membrane potential and an increased mitophagy rate. The appearance of mitochondrial fragmentation during the neuronal differentiation process was preceded by translocation of CL to the OMM in mutant neurons and A53T transgenic mice. Mutant α-Syn also showed a reduced ability to compete with the binding of LC3 to CL at the OMM, which might explain the increased mitophagy rate in mutant neurons (Ryan et al., 2018). By using circular dichroic spectroscopy, the same study found that CL in OMM-mimics is able to promote not only the folding of intrinsically disordered monomers to helical structures but also the refolding of pathological oligomers and fibrils to monomers with a helical conformation by pulling away α-Syn monomers from oligomers and fibrils. The rate of refolding was reduced in oligomers and fibrils of mutant α-Syn compared to wild-type α-Syn (Ryan et al., 2018). However, further research is needed in order to investigate whether such a CL-dependent mechanism to buffer toxic α-Syn species occurs *in vivo*, especially since CL translocation to the OMM might increase the α-Syn/mitochondria interaction, leading to pore formation and mitochondrial damage (Ghio et al., 2019). Therefore, additional studies are needed to determine more precisely, which phenomenon occurs under which circumstances.

In summary, α-Syn species preferentially bind to CL-enriched mitochondrial membrane microdomains. In addition to interaction with mitochondrial proteins, the phospholipid CL is an important mediator for the binding of α-Syn to mitochondria. This binding, together with reduced CL content, altered composition and oxidation as well as its translocation from the IMM to the OMM induce impaired mitochondrial respiration, mitochondrial membrane leakage and excessive mitophagy. However, OMM-localized CL was also shown to refold α-Syn oligomers and fibrils, and it might thus be able to protect from pathogenic effects of α-Syn aggregates. Further studies are needed to determine under which circumstances protective effects of CL – levels and localization – might occur.

## Concluding Remarks

A large amount of evidence collected in the last 20 years highlights the transient binding of α-Syn to lipids and FAs under physiological conditions, while an excessive membrane binding might lead to toxic oligomer formation on the membrane surface and disruption of membrane integrity. Alterations of α-Syn/vesicle binding might lead to an abnormal clustering and accumulation of synaptic vesicles, ultimately impacting the neurotransmitter release into the synaptic cleft. Existing literature indicates increased MUFA and PUFA levels in PD, which both contribute to pathological α-Syn oligomer formation. α-Syn was shown to accumulate at CL-enriched microdomains of mitochondrial membranes and impact mitochondrial function. Binding of α-Syn to mitochondria and altered CL content, composition and oxidation lead to alterations in mitochondrial morphology and membrane integrity culminating in impacted mitochondrial respiration, increased mitophagy, and Cyt c release. CL-mediated binding of α-Syn to mitochondria might thus represent an important mechanism in the pathogenesis of PD. Similar to synaptic vesicles, α-Syn might not only impact mitochondrial integrity but also contribute to the clustering of dysfunctional mitochondrial membranes, potentially leading to the accumulation of fragmented mitochondria in LB-type inclusions. Further studies are needed to determine the mechanistic details and chronological sequence of the α-Syn/CL interaction, the induction of mitochondrial dysfunction, and the role of CL-specific regulatory mechanisms in the refolding of pathological α-Syn species. These studies will help clarify the usefulness of pharmacological interventions to preserve/reconstitute CL content and composition as well as localization in the disease process.

## Author Contributions

IP and ML conceptualized and designed the manuscript. VG and GG drafted the manuscript. AZ, MPCR, AAH, PPP, ML, and IP provided critical revisions of the manuscript. All authors contributed to the article and approved the submitted version.

## Conflict of Interest

The authors declare that the research was conducted in the absence of any commercial or financial relationships that could be construed as a potential conflict of interest.
